# Antioxidant Activity of *Annurca* Apple By-Products at Different Ripening Stages: A Sustainable Valorization Approach

**DOI:** 10.3390/antiox14080941

**Published:** 2025-07-30

**Authors:** Pasquale Perrone, Sara Palmieri, Marina Piscopo, Gennaro Lettieri, Fabiola Eugelio, Federico Fanti, Stefania D’Angelo

**Affiliations:** 1 Department of Medical, Movement, and Wellbeing Sciences (DiSMMeB), Parthenope University of Naples, 80133 Naples, Italy; pasquale.perrone@collaboratore.uniparthenope.it; 2 Department of Biosciences and Technologies for Agriculture, Food and Environment, University of Teramo, Campus di Coste Sant’Agostino, 64100 Teramo, Italy; spalmieri@unite.it (S.P.); feugelio@unite.it (F.E.); ffanti@unite.it (F.F.); 3 Department of Biology, University of Naples Federico II, Via Cinthia, 21, 80126 Naples, Italy; marina.piscopo@unina.it (M.P.); gennaro.lettieri@unina.it (G.L.)

**Keywords:** *Annurca* apple, apple by-products, polyphenols, antioxidant ability, nutraceutical potential, ripening stage, sustainable valorization, agro-industrial waste

## Abstract

This study explores the sustainable valorization of *Annurca* apple by-products by examining the polyphenolic content and antioxidant activity of peel, flesh, and core at two ripening stages. Ripening significantly enhanced the concentration of bioactive compounds, particularly in the peel, where total polyphenols increased from 124.4 to 423.3 mg of CAE/100 g FW, flavonoids from 18.2 to 51.3 mg of quercetin equivalents, and ortho-diphenols from 11.9 to 36.1 mg of CAE. The flesh and core showed more moderate increases. Antioxidant activity, assessed using five in vitro assays (DPPH, ABTS, FRAP, TAC, and H_2_O_2_), was consistently highest in the peel, especially in the ABTS assay. Although the flesh had fewer phenolics, it showed a 1.5-fold increase during ripening, accompanied by improved antioxidant performance. The core also proved notable antioxidant potential, particularly in ripe samples. UHPLC-MS/MS analysis identified 11 phenolic compounds, showing tissue- and ripening-specific distribution. SDS-PAGE revealed a ripening-related increase in Thaumatin-like Protein 1a (TLP1a), especially in the core and flesh. Its association with tissues showing high antioxidant ability suggests a possible role in enhancing the bioactivity of polyphenol-rich extracts. From an agri-food waste valorization perspective, the peel and core represent promising sources of bioactive compounds for developing functional foods and nutraceuticals.

## 1. Introduction

There is a growing demand for bioactive compounds from consumers interested in healthy foods of natural origin, and many fruits are important sources of such compounds with antioxidant ability. Moreover, the increasing focus on environmental sustainability and food waste reduction has encouraged research into innovative strategies for valorizing by-products from the fruit and vegetable industry [1].

In this context, the *Annurca* apple from Campania (*Malus pumila Mill*.), a native cultivar recognized for its excellent nutritional and sensory properties, stands for a resource of particular interest. Specifically, peels and cores, often considered waste, are in fact rich in bioactive compounds such as polyphenols, flavonoids, and pectins, known for their antioxidant and health-promoting properties. The *Annurca* apple is called the “queen of apples” due to its remarkable organoleptic qualities, taste, flavor, and aroma, and it is a key part of the Mediterranean diet.

The *Annurca* apple is a unique *cultivar* in the world due to its traditional post-harvest reddening process. This distinctive trait significantly influences its biochemical composition and sets it apart from all other apple varieties studied to date. In fact, one of the most distinctive features of the *Annurca* apple is its unique ripening process, known as “redding on the ground”. Unlike all apple varieties, *Annurca* does not ripen on the tree: the fruits are harvested while still unripe. This is not simply due to the growers’ caution but rather to a specific botanical trait, the particularly short stem (typically 7 to 14 mm), which is not strong enough to support full ripening on the tree. Harvesting takes place in the last days of September (variable period because it depends on the climate), when the apples are still green with red streaks. After picking, the apples are laid out in the sun to ripen on beds made of wood shavings or pine needles, known as “melai”. To protect them from excessive solar radiation or adverse weather conditions, the “melai” are covered with special sheets or breathable cloths that allow light to pass through. The apples are regularly turned by hand and left to ripen for 20 to 40 days, until the skin turns 90–100% red. It is precisely this “melai” reddening process that elevates the quality of the *Annurca*, endowing it with a unique identity that no other apple variety can match. Its potential health benefits are largely attributed to its high content of bioactive compounds, particularly polyphenols [2].

Polyphenols are a diverse class of plant secondary metabolites renowned for their potent antioxidant activity, which enables them to effectively scavenge free radicals and mitigate oxidative stress in biological systems. Their health-promoting effects are largely attributed to their ability to modulate key cellular signaling pathways, exert anti-inflammatory actions, and confer protection against a range of chronic diseases, including cardiovascular disorders, diabetes, and cancer [3,4,5,6]. This broad group of compounds, including flavonoids, phenolic acids, and tannins, not only contributes to the organoleptic properties of fruits, such as color and flavor but also plays a crucial role in cellular defense mechanisms. The ability of polyphenols to prevent lipid peroxidation and DNA damage underpins their potential application in the development of functional foods and nutraceuticals. Importantly, the bioavailability and biological efficacy of polyphenols are influenced not only by their total concentration but also by their chemical structure, stability, and interactions within the food matrix, which can modulate their absorption and metabolic fate.

Among the main bioactive compounds in *Annurca* apples, polyphenols, especially quercetin, chlorogenic acid, and catechins, stand out for their high ability to neutralize free radicals, reduce oxidative stress, and modulate inflammatory processes at the cellular level. These properties make them highly interesting candidates for the development of functional ingredients and protective health products.

Various studies have proved the antioxidant and pro-oxidant effects of polyphenol extracts from *Annurca* apple flesh on different cell models. The health benefits of *Annurca* apple polyphenols include potential protective effects against obesity, liver disease, alopecia, diabetes, cancer, and cardiovascular diseases. Compared to other fruits, *Annurca* apples generally show a higher content of bioactive compounds, making them ideal for nutraceutical applications. Extracts from *Annurca* apple flesh show strong antioxidant activity in vitro, often surpassing that of other common apple cultivars. This activity is attributed to the synergistic effects of multiple polyphenols [7,8,9]. Additionally, *Annurca* biophenols reduce free radical formation in human erythrocytes [10] by inhibiting the oxidation of low-density lipoproteins, a factor contributing to atherosclerosis development [11].

Beyond antioxidant activity, polyphenolic molecules demonstrate antiproliferative effects, which may inhibit tumor formation and progression. For example, *Annurca* apple flesh polyphenols can slow cell proliferation in human keratinocytes, a predictive model for dermo toxicity screening [12]. This supports the hypothesis of *Annurca* apple’s potential in phototherapy. These polyphenols significantly reduce cell viability in a dose-dependent manner, cause morphological changes, and induce apoptosis via an extrinsic, p53-independent pathway [13]. One study highlighted the antitumor effects of *Annurca* apple polyphenol flesh extract (AAPPE) on triple-negative breast cancer (TNBC) cell lines (MDA-MB-231 and MDA-MB-468). AAPPE selectively reduced TNBC cell viability by inducing G2/M cell cycle arrest and triggering reactive oxygen species (ROS) production in cancer cells while acting as an antioxidant in non-tumorigenic MCF10A cells. Moreover, AAPPE inhibited cell migration and metastasis, positioning it as a promising natural compound for TNBC prevention and treatment via ROS-mediated JNK activation and epithelial-to-mesenchymal transition inhibition [14,15].

Scientific interest in the *Annurca* apple has also increased due to its excellent agronomic traits, long shelf life, and the potential for quality improvement through bio-stimulants and post-harvest innovations. A distinctive feature of the *Annurca* apple is its post-harvest reddening process, which leads to significant changes in phytochemical composition between unripe (green) and ripe (red) fruits. Recent studies suggest that the content and bioavailability of phenolic compounds vary according to the maturation stage, thereby affecting the antioxidant potential of the extracts obtained [16,17]. These characteristics make the *Annurca* apple an ideal model for multidisciplinary studies ranging from agronomy to nutraceuticals, with significant implications for public health and the valorization of local productions.

The phenolic composition of plants is influenced by multiple factors, including genotype, phenological stage, plant tissue, extraction method, environmental conditions, and geographic origin [18,19,20]. These variables need site-specific and methodologically tailored analyses to assess phytochemical potential. However, during industrial processing and post-harvest handling, a significant portion of the fruit, particularly peel and core, is discarded. If properly valorized, these by-products could become valuable resources for the food, cosmetic, and nutraceutical industries. Recently, interest in agro-industrial by-products as alternative sources of bioactive compounds has increased.

This study aims to investigate and valorize by-products derived from the processing of *Annurca* apples, specifically peel and core, obtained from fruits at two ripening stages: unripe and fully ripe. The aims are to assess their polyphenol content, chemically characterize the extracts, and evaluate their antioxidant activity through various in vitro assays. The valorization of these by-products not only helps reduce environmental impacts related to organic waste disposal but also offers a concrete opportunity to recover high-value-added compounds usable in sectors such as functional foods, natural cosmetics, and pharmaceuticals. This approach aligns fully with the principles of the circular economy, promoting a more efficient and sustainable production model.

## 2. Materials and Methods

### 2.1. Fruit Collection

*Annurca* apples (Malus pumila Mill. cv. *Annurca*) were collected in 2024 from an orchard located in Giugliano in Campania (Naples, Italy). Fruits were harvested in September at the pre-climacteric stage, characterized by green peel and incomplete ripening. A subset of these unripe apples was at once processed for analytical purposes. The remaining fruits underwent the traditional post-harvest reddening process in “melai”, raised beds of well-drained soil covered with straw, where apples were exposed to natural sunlight for approximately one month (October). This treatment enhances the typical red coloration and sensory attributes of the cultivar. After the reddening phase, ripe fruit samples were collected and processed for comparative analysis [21].

### 2.2. Chemicals and Solutions

2,2′-diphenyl-1-picrylhydrazyl (DPPH) was from Sigma Chemical Co. (St. Louis, MO, USA). Methanol was from Romil Ltd. (Cambridge, UK). Hydrogen peroxide 30% was from ITW Reagents (Darmstadt, Germany). ABNOKA1622 assay kit was from ABNOVA (Taipei, Taiwan). MAK509 kit was from Sigma-Aldrich (St. Louis, MO, USA). ABTS assay kit (E-BC-K271-M) was from ElabScience (Houston, TX, USA). Electrophoresis reagents and apparatus were from Bio-Rad (Bio-Rad Laboratories S.r.l., Milan, Italy). Catechin, gallic acid, epicatechin, quercetin, caffeic acid, coumaric acid, chlorogenic acid, and cinnamic acid were from Sigma Chemical Co. (St. Louis, MO, USA).

### 2.3. Annurca Apple Samples

**Figure 1:**
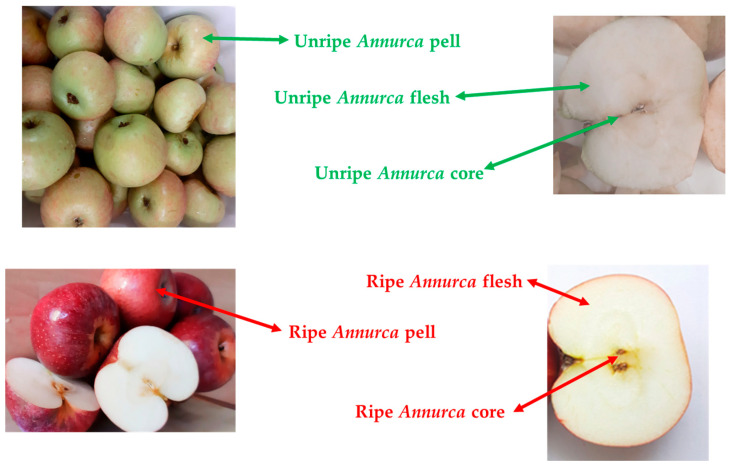
Analyzed parts of *Annurca* apple (Photo by S.D.).

Both unripe and ripe *Annurca* apples (each weighing approximately 100 g) were used for the experiments. For each ripening stage, peel, flesh, and core tissues (Figure 1) were manually separated and analyzed [17].

### 2.4. Polyphenol Extraction

Forty grams of *Annurca* apple sample was homogenized for 5 min by a Tefal rondo 500 homogenizer using 40 mL of 80% methanol and 20% water plus 0.18 N HCl (15 mL 12 N of HCl/L). After centrifugation (18,000× *g* for 25 min), the slurry was dried under vacuum by using the Univapor Concentrator Centrifuge, model Univapo 100 H (Uni Equip, Germany). The dried extracts were dissolved in 10 mL of PBS and frozen at −80 °C until use [17].

### 2.5. Polyphenolic, Flavonoids, and Ortho-Diphenolic Contents

The total polyphenolic content of apple extracts was assessed approximately by using the Folin–Ciocalteu phenol reagent as described in ref. [22,23]. The extracts (100 μL) were mixed with the Folin–Ciocalteu phenol reagent (0.5 mL), deionized water (0.9 mL), and Na_2_CO_3_ (7.5% *w*/*v*, 4 mL). The absorbance at 765 nm was measured 2 h after incubation at room temperature using a Cary ultraviolet–visible spectrophotometer (Varian). The measurement was compared to a standard curve of prepared catechin solutions and expressed as milligrams of catechin (CAE) equivalent per 100 g of FW (Fresh Weight) apple part.

The content of total flavonoids was determined by the AlCl_3_ colorimetric method [24]. Briefly, 80 μL of the sample extract was mixed with 80 μL of 2% AlCl_3_ solution and 120 μL of aqueous sodium acetate solution. The reaction mixture was placed in the dark at 25 °C for 2.5 h, and the absorbance was recorded at 440 nm. The measurement was compared to a standard curve of prepared quercetin solutions and expressed as milligrams of Quercetin equivalent per 100 g of FW (Fresh Weight) apple part.

The *ortho*-diphenolic content was decided calorimetrically using the Arnow reagent [17,23]. Briefly, the samples (400 μL) were mixed with three solutions added in the following order: solution A (0.5 M HCl, 400 μL), solution B (1.45 M NaNO_2_–0.4 M Na_2_MoO_4_, 400 μL), and solution C (1 M NaOH, 400 μL). The absorbance of the sample was measured at 500 nm, and the *ortho*-diphenolic content was compared to a standard curve of prepared catechin solutions and expressed as milligrams of catechin (CAE) equivalent per 100 g of FW (Fresh Weight) apple part.

### 2.6. Ultra-Performance Liquid Chromatography–Electrospray Ionization–Tandem Mass Spectrometry (UPLC-ESI-MS/MS)

An Acquity H-Class chromatographic system (Waters, Milford, MA, USA) was coupled with a QTrap 4500 mass spectrometer (Sciex, Toronto, ON, Canada) according to Oliva et al. [25], with slight modifications. Briefly, each extract was diluted with 1 mL of phosphate buffer (50 mM): MeOH (9:1 *v*/*v*), which is the loading of purification phase with Solid Phase Extraction (SPE). A Strata XL cartridge (330 mg, 1 mL) from Phenomenex (Torrance, CA, USA) was used for clean-up step and then analyzed by UPLC-ESI-MS/MS in Multiple Reaction Monitoring (MRM) acquisition modes operating in negative ionization. For chromatographic separation, an Excel 2 C18-PFP 2.0 µm (100 × 2.1 mm) column was employed; H_2_O 0.5% acetic acid (phase A) and ACN (phase B) were used as mobile phases. The column operated at a flow rate of 0.6 mL/min. The column oven was set at 40 °C. For detection, the MRM acquisition was used; for each PC, at least two MRM transitions were monitored, and each of them was carefully tuned by injection of the correlated analytical standard. Data collection and processing were performed with Analyst 1.7.3 software and quantification with Multiquant 3.0.3 software, both from Sciex.

### 2.7. Determination of Antioxidant Capacity

#### 2.7.1. DPPH Assay

The free radical scavenging capacity of the different apple extracts was assessed using the 2,2′-diphenyl-1-picrylhydrazyl (DPPH) assay, according to the procedure described by Belinky et al. [26]. DPPH reagent was prepared by dissolving 9 mg of DPPH in 100 mL of methanol (0.23 mM). Briefly, 1 mL of each extract was diluted with 2 mL of DPPH. The samples were incubated at room temperature in the dark for 30 min. The absorbance was recorded at 517 nm using the model UV-3100PC spectrophotometer (VWR International, Avantor Group, United States of America). The percentage of inhibition was calculated using the following equation:

$$DPPH.inhibition\left(\%\right)=\frac{Ac-As}{Ac}\;\times 100$$

where Ac is the absorbance of the control (DPPH solution in methanol), and As is the absorbance of the sample.

#### 2.7.2. Hydrogen Peroxide Recovery Assay

The ability of apple extracts to scavenge hydrogen peroxide was detected according to the method described in ref. [27]. Specifically, apple samples (15 μL) were diluted in 2 mL of distilled water and added to 0.3 mL of 4 mM H_2_O_2_ solution. The samples were incubated for 10 min in the dark at room temperature. Then, the H_2_O_2_ concentration was evaluated spectrophotometrically at 230 nm, using the model UV-3100PC spectrophotometer, and the scavenger capacity of the samples was evaluated by comparing it with a blank having the extracts in PBS without H_2_O_2_.

#### 2.7.3. Total Antioxidant Capacity Assay

The total antioxidant capacity (TAC) of the apple extract samples was measured using the ABNOKA1622 assay kit. The procedure followed the manufacturer’s instructions. Specifically, the assay is based on the reduction of Cu^2+^ to Cu^+^ by antioxidants present in the sample. The resulting Cu^+^ forms a colored complex with a proprietary chromogenic reagent. Thus, the assay provides a quantitative colorimetric determination of TAC. Briefly, 20 μL of apple extract samples were mixed with 100 μL of the working solution in wells of a 96-well microplate. The plate was then incubated at room temperature for 10 min, after which absorbance was measured at 570 nm with IMARK microplate reader. TAC values were evaluated using a standard curve generated with Trolox.

#### 2.7.4. FRAP

The MAK509 kit (Sigma-Aldrich, MilliporeSigma, United States of America) was used to evaluate the ferric reducing antioxidant power (FRAP) assay. This test assesses antioxidant potential based on the principle that Fe^3+^ is reduced to Fe^2+^ by antioxidants present in the sample. The resulting Fe^2+^ forms a colored complex with a specific chromogenic reagent. The assay was performed according to the manufacturer’s recommended protocol. Briefly, 50 μL of apple extract samples were mixed with 200 μL of working solution in wells of a 96-well microplate. The plate was then incubated at room temperature for 40 min, after which absorbance was measured at 590 nm, using IMARK microplate reader. FRAP values were calculated using the following equation:

[FRAP](μM Fe^3+^ reduction potential) = Rsample-Rblank/Slope

where Rsample is the absorbance of samples, and Rblank is the absorbance of blank.

#### 2.7.5. ABTS Assay

The ABTS assay was used to assess the TAC of the samples. The principle of the method is based on the oxidation of ABTS to its radical cation (ABTS^+^), which produces a green coloration. In the presence of antioxidants, this oxidation is inhibited, leading to a decrease in absorbance. The assay was performed following the manufacturer’s instructions. Briefly, 10 μL of apple extract samples were mixed with 200 μL of working solution in the wells of a 96-well microplate. The plate was incubated at room temperature for 5 min, and absorbance was measured afterwards at 734 nm, using IMARK microplate reader. TAC values were evaluated using a standard curve generated with Trolox.

### 2.8. SDS-PAGE

SDS-PAGE electrophoretic run was performed using a 1.0 mm gel with 16% Tris-glycine. In total, 4 µg of samples was boiled at 100 °C for 10 min with 10 µL of 1× Laemmli buffer. The electrophoretic run was conducted in Tris-glycine 1× running buffer at 150 V for about 2 h. The gel was stained with both 0.25% Comassie Blue solution in 30% methanol and 10% acetic acid. Finally, the image was obtained with the Biorad GelDoc system, using ImageLab 6.0.1 software (build 34) (BioRad, Hercules, CA, USA).

### 2.9. Statistical Analyses

Data are expressed as means ± standard deviation (S.D.) of three independent experiments. Statistical significance was detected by one-way ANOVA followed by Tukey’s post hoc multiple comparison test. GraphPad Prism 10 was used for statistical analysis.

## 3. Results

### 3.1. Concentration of Phenolics, Flavonoids, and Ortho-Diphenols in Extracts of Annurca Apple

The concentration of the individual phenolic compounds in the apple is not constant. It depends on the cultivar, the maturity of the fruit, the growing conditions, the growth, the harvest, the storage, and the infections suffered. It can be changed by post-harvest factors, including conservation and processing [28]. In general, the chemical profile and its variations are caused by the growing season, geographical location, and, above all, genetic variation [29].

In our study, all apples analyzed belonged to the *Annurca* cultivar and were harvested in the same season from orchards under comparable agronomic and environmental conditions. This allowed us to minimize variability due to external factors and focus on changes related specifically to the ripening stage.

The concentrations of total polyphenols detected in the various extracts of the *Annurca* apple are reported in Table 1. In the unripe apple samples, an uneven distribution of polyphenols among the different fruit tissues was seen. Specifically, the peel showed a significantly higher concentration compared to both the flesh and the core (*p* < 0.001). The flesh, in turn, showed approximately twice the number of polyphenols as the core (*p* < 0.001).

In the ripe apple samples, a markedly different pattern in the distribution of polyphenols was seen. The peel had a polyphenol content approximately five times higher than that of the flesh and about seven times higher than that of the core (*p* < 0.001). Moreover, when comparing the peel of ripe apples to that of unripe apples, an approximately 4-fold increase in total polyphenol content was detected (*p* < 0.001), showing significant accumulation during the reddening and ripening process.

From a practical perspective, such a 4-fold increase may have relevant implications in terms of biological activity, considering that polyphenols are known for their antioxidant, anti-inflammatory, and potential health-promoting properties. Therefore, the more mature peel could be a more valuable source of functional compounds for nutraceutical or food applications.

This increase may be attributed to the action of ethylene, a key hormone in climacteric fruit ripening, known to stimulate the activity of the enzyme phenylalanine ammonia-lyase, which plays a central role in the biosynthesis of phenolic compounds. The characteristic red color of the apple peel is due to the accumulation in the vacuoles of cyanidin 3-galactoside, an *o*-diphenol belonging to the anthocyanin class.

About the other tissues, the flesh also showed an increase in polyphenol content during ripening, with an approximate 1.5-fold increase (*p* < 0.01), while, in the core, the increase was approximately 3-fold (*p* < 0.001). Overall, the data show that the ripening process leads to an increase in polyphenol content in all parts of the fruit, although this increase is more pronounced in the peel and core than in the flesh.

Given that the flesh represents about 85% of the edible part of the fruit, even a moderate increase in polyphenol content during ripening can contribute substantially to the overall intake of these compounds through diet.

Flash represents about 85% of the weight of the fruits (edible part); it has a high polyphenolic content, already present in the flesh obtained from the unripe fruit just harvested, therefore prior to the ripening process in the *melai*.

Regarding the total flavonoid content, the data are reported in Table 1. As can be evaluated, during the ripening process, there is an increase of approximately 50% in the flavonoid content of the flesh. In contrast, the peel shows a more pronounced increase, about 2.5 times higher.

This significant increase in the peel may further enhance the antioxidant capacity of the fruit and suggests its potential value for the development of functional food ingredients or supplements.

Interestingly, no significant differences are detected in the core between ripe and unripe apple samples. It is also worth noting that, as previously evaluated for the total phenolic content, the peel is again the part with the highest concentration of flavonoids. Specifically, in unripe apple samples, the flavonoid concentration in the peel is about 10 times higher than in the flesh and core, while, in ripe apples, the increase reaches approximately 25 times.

As shown in Table 1, the *ortho*-diphenol content increases during ripening in all parts of the fruit. The most pronounced change is evaluated in the peel, where the concentration rises from 11.9 ± 1.9 in unripe apples to 36.1 ± 2.5 in ripe ones, which is an approximate 3-fold increase. In the flesh, the content increases modestly from 2.0 ± 1.2 to 2.8 ± 1.3, while the core shows a similar trend, with values rising from 2.4 ± 0.7 to 3.7 ± 1.1. These results confirm that the peel is the richest source of *ortho*-diphenols, both in unripe and ripe stages, and suggest that the ripening process markedly enhances the accumulation of these compounds, especially in the external tissues of the fruit.

Such increases may be particularly relevant for applications in the nutraceutical sector, as ortho-diphenols have been associated with various beneficial biological effects, including antioxidant and cardioprotective properties.

### 3.2. UHPLC-MS/MS Analysis

A total of fourteen phenolic compounds (catechin, epicatechin, quercetin, quercetin hexoside, 4-hydroxybenzoic acid, protocatechuic acid, OH-tyrosol, vanillic acid, caffeic acid, ferulic acid, chlorogenic acid, rutin, synaptic acid, *p*-*o*-coumaric acid) were evaluated, primarily belonging to the classes of phenolic acids and flavonoids. UHPLC-MS/MS analysis revealed differences in phenolic compounds among the various parts of the *Annurca* apple (peel, flesh, and core) and between unripe and ripe stages.

**Figure 2:**
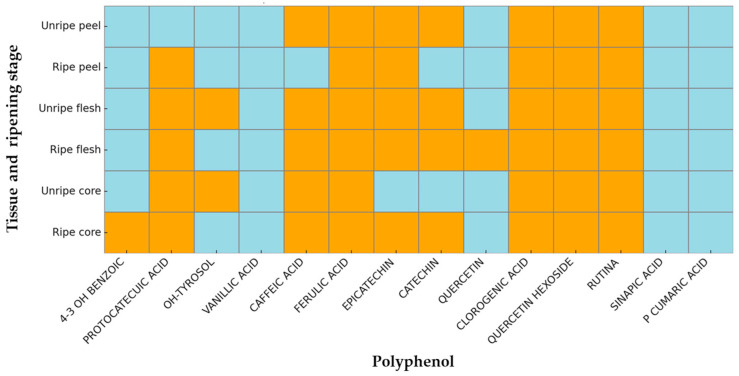
Binary heat map showing the presence (orange) or absence (light blue) of selected polyphenolic compounds across different fruit tissues (peel, flesh, core) and ripening stages (unripe vs. ripe). Rows are tissue types at specific maturity stages, while columns correspond to individual polyphenols. The map highlights distinct tissue- and maturity-dependent patterns in polyphenol occurrence. Compounds labeled as “LOQ” (Limit of Quantification) data are encoded as “0” (absence) in the binary matrix.

The binary heat map analysis (Figure 2) revealed distinct patterns in polyphenol presence across different tissue types and ripening stages. Notably, in unripe samples, key polyphenols such as epicatechin, catechin, chlorogenic acid, and quercetin hexoside were consistently detected, particularly in peel and flesh. This suggests their involvement in early-stage defense and developmental processes. In contrast, ripe samples showed the presence of compounds not detectable in immature tissues, such as 4-hydroxy-3-benzoic acid and quercetin, showing possible induction during ripening or senescence-related metabolic shifts. Vanillic acid and *p*-coumaric acid were absent across all samples, suggesting either concentrations below detection thresholds or a lack of biosynthetic activity under the studied conditions. The ripe flesh displayed the highest diversity of polyphenols, including both flavonoids and phenolic acids, while ripe peel showed a comparatively reduced profile.

These observations highlight the maturity-dependent modulation of the polyphenolic composition, which appears to be tissue-specific and likely reflects underlying physiological and biochemical transitions associated with fruit development and ripening [30,31,32].

### 3.3. Changes in Radical Scavenging Capacity During Annurca Apple Ripening

#### 3.3.1. DPPH Assay

**Figure 3:**
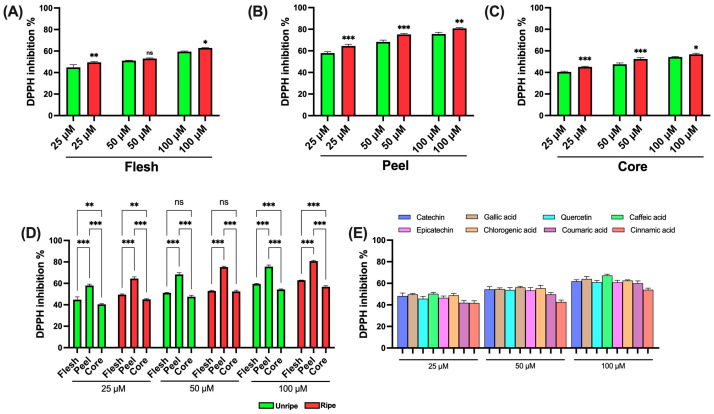
DPPH assay of ripe and unripe *Annurca* apple samples: (**A**) Flesh samples. (**B**) Peel samples. (**C**) Core samples. (**D**) Differences between samples of the different parts. (**E**) Standards for positive control. Statistical analysis is performed using one-way ANOVA followed by Tukey’s test. *** (*p* < 0.001), ** (*p* < 0.01), * (*p* < 0.05), and ns (*p* > 0.05).

The antioxidant activity of various apple extracts was found using established spectrophotometric assays to ensure correct and comparable evaluation. The use of different methods is essential for a comprehensive characterization of the antioxidant potential of the samples analyzed. Initially, the DPPH assay was performed, based on the reduction in the stable free radical 2,2-diphenyl-1-picrylhydrazyl (DPPH•), which results in a measurable decrease in absorbance. DPPH is commonly used to assess the antioxidant activity of natural compounds. Antioxidants interact with DPPH by donating an electron or a hydrogen atom, thus neutralizing the radical. The results shown in Figure 2 show that all tested extracts were capable of significantly reducing the DPPH• radical. Specifically, flesh extracts displayed only minor differences between ripe and unripe apples across all tested concentrations (Figure 3A). In contrast, peel extracts showed increased antioxidant activity in samples from ripe apples at all concentrations analyzed (Figure 3B). A similar trend was evaluated in the core extracts, where samples from ripe apples showed higher antioxidant activity (Figure 3C). Figure 3D presents a comparison among the different parts of the apple. Peel extracts prove significantly higher antioxidant activity compared to both flesh and core, across all tested concentrations and for both ripeness stages. The flesh extracts showed higher activity than the core extracts only at low (25 μM) and high (100 μM) concentrations. Finally, Figure 3E shows the data obtained from the standard compounds used as positive controls. All standards displayed a similar antioxidant profile across the tested concentrations. However, caffeic acid showed the highest antioxidant ability, while cinnamic acid showed the lowest.

Figure S1 shows the IC_50_ values of the different components of the *Annurca* apple. In particular, the data show that the components of the ripe apple exert a greater effect. In fact, the concentration needed to achieve 50% inhibition is lower in all three cases.

It is also interesting to note that the peel shows a significantly stronger effect compared to the other components. Specifically, the concentration needed to achieve 50% of the effect is about three times lower than that of the flesh and four times lower than that of the core. Figure S1 also reports the IC_50_ values of the standards used as positive controls. The greatest effect is seen as caffeic acid, while the weakest effect is seen as cinnamic acid.

#### 3.3.2. Hydrogen Peroxide Recovery Assay

**Figure 4:**
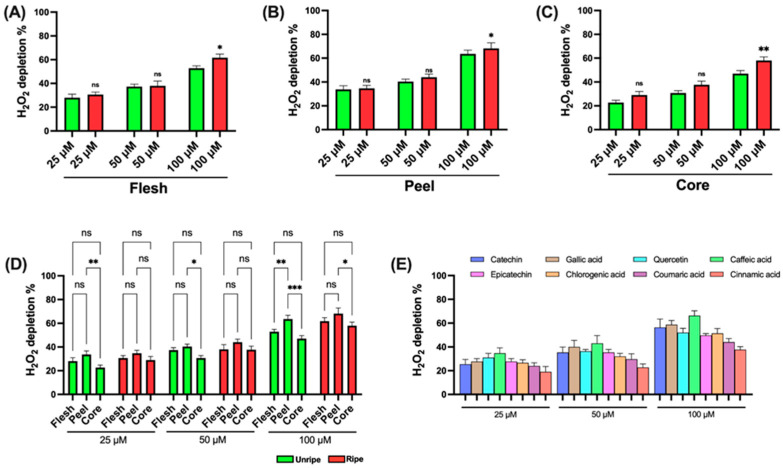
H_2_O_2_ depletion assay of ripe and unripe *Annurca* apple samples: (**A**) Flesh samples. (**B**) Peel samples. (**C**) Core samples. (**D**) Differences between samples of the different components. (**E**) Standards for positive control. Statistical analysis is performed using one-way ANOVA followed by Tukey’s test. *** (*p* < 0.001), ** (*p* < 0.01), * (*p* < 0.05), and ns (*p* > 0.05).

Figure 4 shows the data on the H_2_O_2_ depletion capacity of the various components of the *Annurca* apple. The assay used to evaluate antioxidant ability is based on the ability of the sample to neutralize H_2_O_2_: a lower amount of residual H_2_O_2_ indicates a higher antioxidant activity.

As shown in the figure, statistically significant differences between the components of ripe and unripe apples are detected only at higher concentrations (100 μM) across all three components analyzed (Figure 4A–C). In Figure 4D, statistically significant differences are noted between the flesh and core at 25, 50, and 100 μM in the unripe apple and at 100 μM in the ripe apple. Additionally, a difference is detected between peel and flesh in the unripe apple but only at the highest concentration (100 μM). Finally, Figure 4E reports the data for the standard compounds used as positive controls. In this case as well, caffeic acid shows the strongest effect, while cinnamic acid shows the weakest.

Figure S2 presents the IC_50_ values from the H_2_O_2_ depletion assay. The results show that the peel samples of unripe apples require a concentration approximately 1.5 times lower than the flesh samples and about 2 times lower than the core samples to achieve 50% inhibition.

Similarly, in the ripe apple samples, the peel also shows a higher inhibitory effect, although it is less pronounced than that detected in the unripe apple samples. Nevertheless, across all three components, the ripe apple samples display a more pronounced inhibitory activity compared to those derived from unripe apples. Figure S2 reports the data obtained for the standard compounds employed as positive controls. The results are consistent with earlier observations: caffeic acid shows the most potent inhibitory effect among all standards tested, while cinnamic acid shows the least activity.

#### 3.3.3. Total Antioxidant Capacity Assay

**Figure 5:**
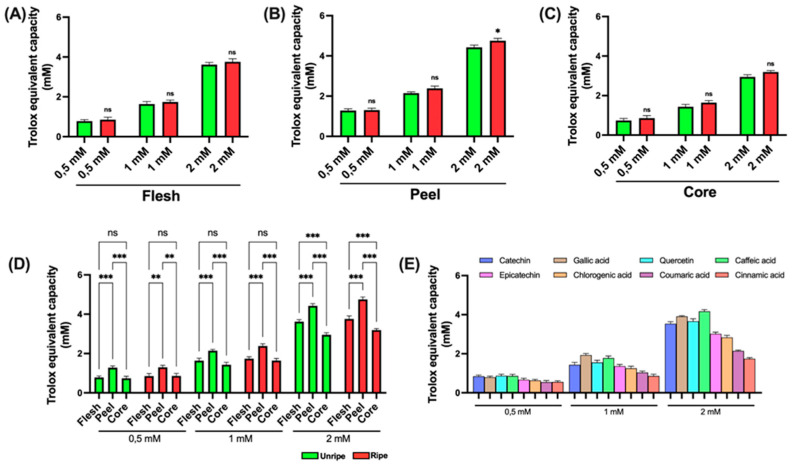
TAC assay of ripe and unripe *Annurca* apple samples: (**A**) Flesh samples. (**B**) Peel samples. (**C**) Core samples. (**D**) Differences between samples of the different components. (**E**) Standards for positive control. Statistical analysis is performed using one-way ANOVA followed by Tukey’s test. *** (*p* < 0.001), ** (*p* < 0.01), * (*p* < 0.05), and ns (*p* > 0.05).

Figure 5 shows the data obtained from the TAC analysis of *Annurca* apple components. As previously described, the assay is based on the reduction of Cu^2+^ to Cu^+^ by antioxidants present in the sample. The resulting Cu^+^ forms a colored complex with a proprietary chromogenic reagent, allowing for a quantitative colorimetric determination of TAC.

As shown in Figure 5A–C, a statistically significant difference between ripe and unripe apple samples is detected only in the peel at high concentrations (2 mM); no relevant differences are detected in the other components. In Figure 5D, a statistically significant difference among peel, flesh, and core is noted across all tested concentrations. Furthermore, at the highest concentration (2 mM), a significant difference between flesh and core becomes clear in both ripe and unripe apple samples. Figure 5E presents the results for the standard compounds used as positive controls. Notably, at the lowest concentration tested (0.5 mM), the various standards show relatively comparable antioxidant activities. In contrast to earlier data, at the intermediate concentration (1 mM), gallic acid proves to have the highest antioxidant effect. At the highest concentration (2 mM) and consistent with earlier findings, caffeic acid shows the most pronounced activity.

Across all concentrations, cinnamic acid consistently shows the lowest antioxidant potential. Figure S3 shows the EC_50_ values associated with the different components of the *Annurca* apple. As illustrated in panels A, B, and C, no significant differences are evaluated between the mature and immature apple samples. Once again, the lowest concentrations needed to achieve 50% of the effect are associated with the peel samples. Figure S3 also presents the data for the reference standards. Caffeic acid and gallic acid display lower and relatively similar EC_50_ values, while the other standards show comparable but slightly higher values. Notably, *p*-coumaric acid and cinnamic acid require substantially higher concentrations, approximately 2.5 and 3.5 times greater, respectively, to reach 50% of the effect.

#### 3.3.4. FRAP

**Figure 6:**
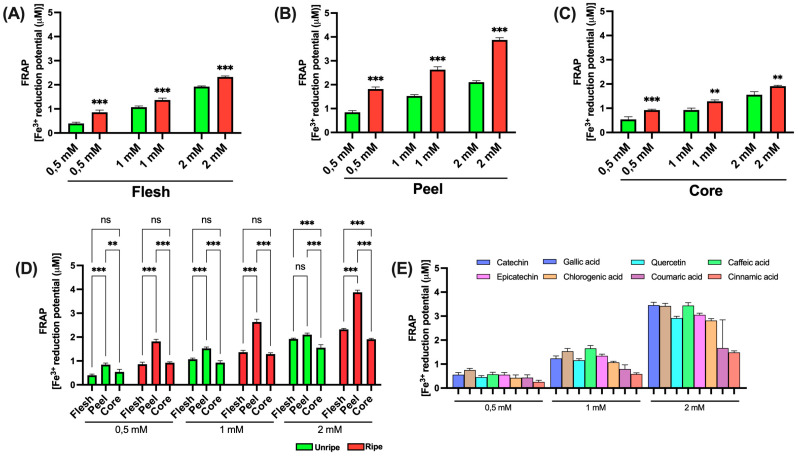
FRAP assay of ripe and unripe *Annurca* apple samples: (**A**) Flesh samples. (**B**) Peel samples. (**C**) Core samples. (**D**) Differences between samples of the different components. (**E**) Standards for positive control. Statistical analysis is performed using one-way ANOVA followed by Tukey’s test. *** (*p* < 0.001), ** (*p* < 0.01) and ns (*p* > 0.05).

The FRAP assay is a spectrophotometric technique commonly used to assess the total antioxidant ability of a sample. The method is based on the reduction of the ferric (Fe^3+^)–TPTZ (2,4,6-tripyridyl-s-triazine) complex to its ferrous (Fe^2+^)–TPTZ form by antioxidants present in the sample, under acidic conditions. This reaction results in the formation of a blue-colored complex, the intensity of which, measured at 593 nm, is directly proportional to the reducing power of the sample. As shown in Figure 6 (panels A, B, and C), statistically significant differences were seen between mature and immature apple samples for all three components analyzed, at all tested concentrations. Data presented in panel D further reveal a significant difference between peel samples and those of flesh and core, both in mature and immature apples. An exception was noted in immature apples at a concentration of 2 mM, where no significant difference was seen between peel and flesh. Consistent with earlier results, a significant difference between flesh and core was also found in both mature and immature apples but only at the highest concentration tested (2 mM). Finally, the data related to reference standards, shown in Figure 6E, show that caffeic acid shows the highest antioxidant activity, except at 2 mM, where its effect is comparable to that of catechin and gallic acid. In line with earlier findings, cinnamic acid showed the lowest antioxidant activity.

The EC_50_ values obtained from the FRAP assay, presented in Figure S4, clearly support the previously described findings. A marked difference is detected between the mature and immature apple samples. Peel samples show significantly lower EC_50_ values compared to flesh and core, which are approximately 0.5-fold and 1-fold lower, respectively. The same figure shows the data for the reference standards. Once again, cinnamic acid displays the lowest antioxidant activity, while the remaining standards show relatively similar effects, with only minor variations among them.

#### 3.3.5. ABTS Assay

**Figure 7:**
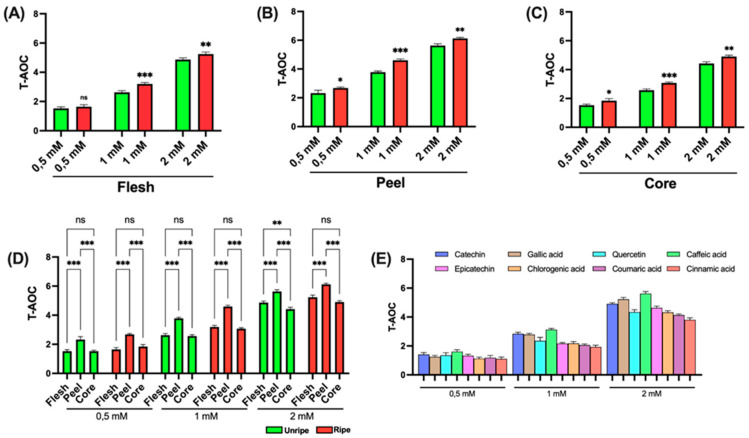
ABTS assay of ripe and unripe *Annurca* apple samples. (**A**) Flesh samples. (**B**) Peel samples. (**C**) Core samples. (**D**) Differences between samples of the different components. (**E**) Standards for positive control. Statistical analysis is performed using one-way ANOVA followed by Tukey’s test. *** (*p* < 0.001), ** (*p* < 0.01), * (*p* < 0.05), and ns (*p* > 0.05).

The ABTS assay is a spectrophotometric method commonly used to evaluate antioxidant ability. The principle of the assay involves the generation of the ABTS^+^ radical cation through the oxidation of ABTS by an oxidizing agent. The resulting green-blue chromophore is after reduced by antioxidants present in the sample, leading to a decrease in absorbance measured at 734 nm. The extent of absorbance reduction is directly proportional to the antioxidant ability of the sample. Figure 7 presents the data obtained from *Annurca* apple samples. As shown in panels A, B, and C, statistically significant differences are not between mature and immature apple samples for all three components analyzed. While significant differences are clear at all tested concentrations for peel and core, in the case of flesh, significance is only found at intermediate and high concentrations (1 mM and 2 mM). Panel D displays data comparing the different apple components. Peel consistently shows greater antioxidant ability compared to both flesh and core, across all tested concentrations and in both pre- and post-ripening samples. Notably, a statistically significant difference between flesh and core is seen at the highest concentration (2 mM), but only in immature apple samples. Finally, the results related to the reference standards, shown in Panel E, show that, consistent with earlier findings, caffeic acid shows the highest antioxidant activity, while cinnamic acid shows the lowest. However, in this assay, the difference between compounds appears less pronounced than in the other tests.

The EC_50_ values obtained from the ABTS assay confirm marked differences between mature and immature apple samples across all three components analyzed. Peel samples show higher antioxidant ability compared to flesh and core, which, in contrast, show relatively similar values. Finally, the standard data, presented in Figure S5, further support earlier findings, showing that caffeic acid shows the highest antioxidant activity, while the lowest activity is associated with cinnamic acid.

### 3.4. SDS-PAGE

**Figure 8:**
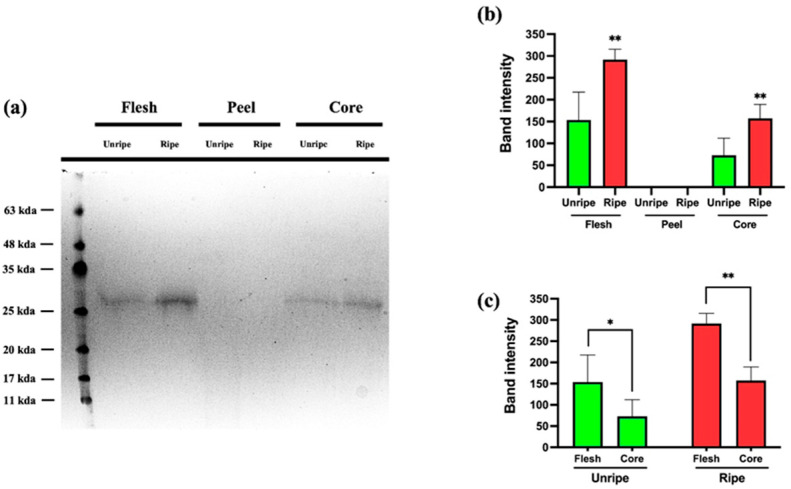
SDS-page analysis of flesh, peel, and core samples of ripe and unripe *Annurca* apples: (**A**) SDS-page. (**B**) Quantification of the band intensity compared to the various samples. (**C**) Statistical difference between flesh and core samples. ** (*p* < 0.01), * (*p* < 0.05).

The SDS-PAGE analysis shown in Figure 8 reveals a band corresponding to a molecular weight of approximately 27–28 kDa. In an earlier study [33], this band was identified as Thaumatin-like Protein 1a (TLP1a). We investigated whether the concentration of this protein varied among different components of the *Annurca* apple at both mature and immature stages. As shown in Figure 8A, the band was detected exclusively in the flesh and core samples, while it was completely absent in the peel, regardless of the ripening stage. Panel B presents the quantification of band intensity. The graph clearly proves a marked increase in Thaumatin-like Protein 1a levels during ripening in both the flesh and core. Finally, the data shown in Panel C indicate that the highest concentration of the protein is found in the flesh, in both mature and immature apple samples.

## 4. Discussion

### 4.1. Polyphenolic Changes During Ripening

In this study, samples were analyzed in their fresh form without prior drying, and the results are expressed as mg GAE per gram of Fresh Weight (FW). This approach reflects a realistic industrial scenario, where polyphenol extraction may occur directly from fresh apple processing by-products. While expressing results on a dry weight basis is common in the literature, we believe that using Fresh Weight offers a more practical and application-oriented perspective.

The results obtained in this study confirm that the ripening of the *Annurca* apple leads to significant changes in the composition and antioxidant ability of the various fruit’s tissues. In particular, the peel proved to be the richest part in polyphenols, with content that increases approximately 4-fold from the unripe to the ripe stage. This increase is consistent with the known role of ethylene in the ripening of climacteric fruits, which stimulates the biosynthesis of phenolic compounds through the activation of the enzyme phenylalanine ammonia-lyase. Moreover, the characteristic red coloration of the ripe peel is attributable to the accumulation of anthocyanins, particularly cyanidin 3-galactoside, which also contributes to antioxidant activity.

The ripening process of the *Annurca* apple is accompanied by a substantial reshaping of the phenolic profile, with increasing total polyphenols, as also reported in other traditional apple cultivars where maturation and post-harvest reddening trigger secondary metabolite biosynthesis [34]. The shift in composition may reflect enzyme-mediated transformations or redistribution of phenolics aligned with physiological needs for protection or storage. Interestingly, similar data were also seen for the profile of flavonoids and ortho-diphenols. These increases may be particularly relevant for nutraceutical applications, as flavonoids and ortho-diphenols have been associated with several beneficial biological effects, including antioxidant and cardioprotective properties. Overall, the findings reinforce that *Annurca* apple by-products, particularly peel and core, are rich sources of antioxidant polyphenols, supporting their potential use as nutraceutical ingredients, a conclusion aligned with in vitro bioactivity assays showing significant antioxidant and enzyme-inhibitory effects [35].

### 4.2. Antioxidant Capacity of Apple Tissues

Spectrophotometric analyses conducted using DPPH, H_2_O_2_ depletion, TAC, FRAP, and ABTS assays provided a comprehensive overview of the antioxidant potential of the different extracts. In all tests, the peel showed significantly higher antioxidant ability compared to the flesh and core, in both unripe and ripe samples. This result was also confirmed by the IC_50_ and EC_50_ values, which were lower for the peel, showing greater effectiveness in neutralizing free radicals and reducing oxidative compounds.

The flesh, although holding fewer phenolics than the peel, showed a significant increase during ripening (approximately 1.5 times), accompanied by an improvement in antioxidant activity. This suggests that the flesh also actively contributes to the fruit’s antioxidant defense mechanisms, albeit with lower efficacy. In the DPPH and ABTS tests, the flesh showed an intermediate response between peel and core, with significant differences especially at the lowest and highest concentrations. This behavior may reflect a more homogeneous but less concentrated distribution of phenolic compounds within the flesh matrix, which is the main edible part of the fruit and is therefore of greater nutritional interest.

The core, often considered a waste product, proved to have notable antioxidant potential, especially in ripe samples. Data showed an approximately 3-fold increase in phenolic content from unripe to ripe fruit, suggesting that this part also takes part in the biochemical processes associated with ripening. Although the total polyphenol content in the core is lower than in the peel and flesh, its antioxidant activity was still significant, as shown by the assays performed.

From the perspective of agri-food waste valorization, these findings are particularly relevant. Peel and core represent potential sources of bioactive compounds that could be exploited to produce functional ingredients, supplements, or natural extracts. These applications, however, should be further confirmed by added research, particularly concerning bioavailability, safety, and efficacy in vivo.

### 4.3. Expression and Potential Role of TLP1a

The SDS-PAGE analysis confirmed the presence of a protein band at ~27–28 kDa, previously identified as Thaumatin-like Protein 1a (TLP1a) in *Annurca* apple tissues [33]. Interestingly, this protein was detected exclusively in the flesh and core, with no expression seen in the peel at either ripening stage. This suggests a tissue-specific distribution of TLP1a, potentially reflecting distinct physiological roles, such as internal defense or stress response mechanisms localized to the fruit’s inner compartments. The progressive increase in band intensity during maturation shows a ripening-associated upregulation of TLP1a. This trend is consistent with earlier findings reporting enhanced expression of TLPs during fruit development and senescence, likely due to their role in pathogen resistance, osmotic regulation, and cellular stabilization. Moreover, the highest levels of TLP1a were evaluated in the flesh, both in unripe and ripe fruits, which may have implications for the nutritional or functional properties of apple-derived products. Given the known bioactivity of thaumatin-like proteins, including antifungal, sweet-tasting, and immunomodulatory effects, these findings could support future applications of *Annurca* apple by-products in the development of functional foods or nutraceuticals.

Although the role of TLP1a in antioxidant defense is not yet fully elucidated, earlier studies have reported its upregulation under abiotic stress conditions, such as drought or salinity, where ROS accumulate [36]. This could suggest a potential involvement in oxidative stress responses, either through direct ROS interaction or through indirect modulation of antioxidant systems.

The presence of TLP1a specifically in tissues that also showed high antioxidant potential further supports the hypothesis that this protein might contribute, directly or indirectly, to the functional performance of polyphenol-rich extracts. TLP1a could potentially interact with phenolic compounds through protein–polyphenol binding mechanisms, influencing their solubility, stability, or bioavailability in formulated products. Such interactions have been documented in other food systems, where TLPs bind polyphenols forming soluble or insoluble complexes [37]. In addition, its possible role as a carrier or structural stabilizer might enhance the shelf life or activity of antioxidant extracts under stress conditions (e.g., temperature, pH, oxidation).

From an applied perspective, understanding the behavior of TLP1a in fruit matrices is crucial for improving extraction processes, especially when targeting both antioxidant and protein fractions for synergistic effects in nutraceuticals or food formulations. Furthermore, since TLPs are also associated with allergenic responses in sensitive individuals, their quantification and tissue distribution are essential for the safe use of apple by-products in health-oriented applications.

Further studies involving proteomic and transcriptomic profiling would be beneficial to elucidate the regulatory pathways underlying TLP1a expression and its functional significance during ripening. Investigating whether TLP1a modulates antioxidant mechanisms or contributes to the stability and efficacy of polyphenol-rich extracts may offer new perspectives for the valorization of *Annurca* apple by-products in sustainable and functional product development.

### 4.4. Tissue-Specific Impact of Ripening

Overall, the data supports the idea that all components of the *Annurca* apple, including by-products such as peel and core, can be valorized within a circular economy framework, contributing to waste reduction and the development of new high-value products. The comparison between ripe and unripe samples revealed significant differences in both phenolic composition and antioxidant ability. In all analyzed tissues, ripening led to an increase in total polyphenol content, with more pronounced effects in the peel and core. This increase was accompanied by improved antioxidant activity, as proven by the reduction in IC_50_ and EC_50_ values across the various assays.

However, the extent of the increase varied among tissues: approximately 4-fold in the peel, 1.5-fold in the flesh, and 3-fold in the core. These differences suggest that the biochemical response to ripening is not uniform but depends on the physiological function and structure of each tissue. From a functional standpoint, ripe samples show greater antioxidant efficacy, making them particularly interesting for nutraceutical and industrial applications. Nevertheless, unripe samples, despite having lower activity, still support a relevant antioxidant profile, which could be useful in contexts where a more moderate phenolic content or a milder sensory profile is desired.

### 4.5. Environmental and Industrial Implications

While the results suggest promising potential for the use of apple by-products, these conclusions must be interpreted with caution. In terms of environmental sustainability, the results of this study highlight the importance of valorizing by-products such as peel and core, generally considered waste, as valuable sources of bioactive compounds. Their use for the extraction of functional ingredients allows for the optimization of natural resource use and the creation of added value from low-cost materials that would otherwise have a high environmental impact if conventionally disposed of. In this context, the *Annurca* apple proves to be a strategic raw material, not only for its organoleptic and nutritional qualities but also for its potential for full use. Unlike other more extensively studied apple cultivars, the *Annurca* is unique in the world due to its traditional post-harvest reddening process, which significantly influences its polyphenolic profile. To date, limited information is available on the valorization of its by-products, especially in relation to ripening stages. The present study contributes novel data on this underexplored cultivar, offering new insights relevant for both scientific understanding and industrial application. The use of natural extracts obtained from by-products such as peel and core may also contribute to the replacement of synthetic antioxidants in food, cosmetic, and pharmaceutical products, responding to the growing demand for natural, safe, and sustainable ingredients.

However, the use of these extracts as direct replacements for synthetic antioxidants in food, cosmetic, or pharmaceutical applications cannot be fully justified based solely on in vitro results. Further toxicological, application, and formulation studies are necessary to confirm their safety and effectiveness under real-world conditions. In addition, some limitations of the present study should be acknowledged. The analyses were conducted exclusively in vitro and therefore do not provide information on the bioavailability, metabolism, or actual physiological effects of the compounds in vivo. Moreover, the number of biological replicates was limited, and the potential influence of seasonal or inter-annual variability was not considered. Furthermore, no toxicological or sensory evaluations were performed to confirm the safety and acceptability of using apple by-products in food applications. These aspects should be addressed in future studies to improve the translational relevance and applicability of the findings.

## 5. Conclusions

This study conducted on the *Annurca* apple highlighted how ripening significantly affects the phenolic composition and antioxidant ability of the fruit’s various tissues. In particular, the peel was confirmed to be the richest in polyphenols and showed the highest antioxidant activity, followed by the flesh and the core. The latter, often considered a waste product, showed notable bioactive potential, especially in ripe samples. Interestingly, even the unripe flesh, despite its lower phenolic content, supported a relevant antioxidant profile, suggesting its potential use in applications where a milder sensory impact or a more moderate phenolic load is desirable. The results emphasize the importance of considering the entire fruit, including by-products, as a functional and sustainable resource. The valorization of peel and core is a concrete opportunity for the agri-food and nutraceutical industries, in line with the principles of circular economy and waste reduction. Looking ahead, the use of natural extracts derived from these tissues could contribute to the replacement of synthetic antioxidants in various sectors, meeting the growing demand for natural, safe, and sustainable ingredients. The *Annurca* apple thus proves to be not only an excellent source in terms of organoleptic and nutritional qualities but also a strategic raw material for the development of new high-value products.

## Figures and Tables

**Table 1:** Polyphenol, flavonoid, and *ortho*-diphenol contents in different parts of the *Annurca* apple, during the ripening process.

*Annurca* Apple Part	Total Polyphenol Content (CAE mg Eq/100 g of FW Sample)		Total Flavonoid Content (Quercetin mg Eq/100 g FW Sample)		Total *Ortho*-Diphenol Content (CAE mg Eq/100 g of FW Sample)	
	Unripe	Ripe	Unripe	Ripe	Unripe	Ripe
Flesh	53.4 ± 2.4	87.3 ± 6.4 **	1.6 ± 0.8	2.3 ± 1.4 *	2.0 ± 1.2	2.8 ± 1.3
Peel	124.4 ± 3.5	423.3 ± 5.8 ***	18.2 ± 2.1	51.3 ± 2.5 **	11.9 ± 1.9	36.1 ± 2.5 **
Core	25.0 ± 1.6	69.5 ± 4.5 ***	2.8 ± 0.6	2.9 ± 0.5	2.4 ± 0.7	3.7 ± 1.1 *

Statistical analysis is performed using one-way ANOVA followed by Tukey’s test. *** (*p* < 0.001), ** (*p* < 0.01), * (*p* < 0.05).

## Data Availability

Data is contained within the article and Supplementary Materials.

## Supplementary Materials

The following supporting information can be downloaded at https://www.mdpi.com/article/10.3390/antiox14080941/s1, Figure S1. IC50 of DPPH assay of ripe and unripe *Annurca* apple samples: (Top) Flesh samples, Peel samples, and Core samples. (Bottom) Standards for positive control. Figure S2. IC50 of H2O2 depletion assay of ripe and unripe *Annurca* apple samples. (Top) Flesh samples, Peel samples, and Core samples. (Bottom) Standards for positive control. Figure S3. IC50 of TAC assay of ripe and unripe *Annurca* apple samples: (Top) Flesh samples, Peel samples, and Core samples. (Bottom) Standards for positive control. Figure S4. IC50 of FRAP assay of ripe and unripe *Annurca* apple samples: (Top) Flesh samples, Peel samples, and Core samples. (Bottom) Standards for positive control. Figure S5. IC50 of ABTS assay of ripe and unripe *Annurca* apple samples: (Top) Flesh samples, Peel samples, and Core samples. (Bottom) Standards for positive control.

## Abbreviations

The following abbreviations are used in this manuscript:

DPPH	2,2′-diphenyl-1-picrylhydrazyl
FRAP	Ferric Reducing Antioxidant Power
TAC	Total Antioxidant Capacity
TLP1a	Thaumatin-like Protein 1a.
UPLC-ESI-MS/MS	Ultra-Performance Liquid Chromatography–Electrospray Ionization–Tandem Mass Spectrometry
