# Supplementary material for: Antioxidant Activity of Annurca Apple By-Products at Different Ripening Stages: A Sustainable Valorization Approach
Source: Antioxidants (Basel). 2025 Jul 30;14(8):941. doi: 10.3390/antiox14080941 (PMC12382625; doi:10.3390/antiox14080941)
Supplement: Supplementary file 1 [file antioxidants-14-00941-s001.zip › antioxidants-3756533-supplementary.pdf]

	IC50 DPPH Assay	
	Unripe	Ripe
Flesh	42,20 μM	29,46 μM
Peel	14,15 μM	9,37 μM
Core	64,69 μM	41,92 μM

	IC50 DPPH Assay
Catechin	30,63 μM
Quercetin	36,27 μM
Caffeic Acid	26,29 μM
Epicatechin	35,26 μM
Cholorgenic Acid	28,52 μM
Coumaric Acid	47,88 μM
Cinnamic Acid	77,3 μM

Figure S1. IC50 of DPPH assay of ripe and unripe *Annurca* apple samples. (Top) Flesh samples, Peel samples and Core samples. (Bottom) Standards for positive control.

	IC50 H_2O_2 Depletion Assay	
	Unripe	Ripe
Flesh	89,63 μM	67,55 μM
Peel	61,27 μM	52,78 μM
Core	120,15 μM	75,16 μM

	IC50 H_2O_2 Depletion Assay
Catechin	81,61 μM
Quercetin	96,74 μM
Caffeic Acid	55,15 μM
Epicatechin	106,8 μM
Cholorgenic Acid	102,3 μM
Coumaric Acid	151,7 μM
Cinnamic Acid	211,4 μM

Figure S2. IC50 of H_2O_2 depletion assay of ripe and unripe *Annurca* apple samples. (Top) Flesh samples, Peel samples and Core samples. (Bottom) Standards for positive control.

	EC50 TAC Assay	
	Unripe	Ripe
Flesh	1,315 mM	1,247 mM
Peel	0,9855 mM	0,9074 mM
Core	1,640 mM	1,456 mM

	EC50 TAC Assay
Catechin	1,387 mM
Quercetin	1,316 mM
Caffeic Acid	1,162 mM
Epicatechin	1,612 mM
Cholorgenic Acid	1,752 mM
Coumaric Acid	2,490 mM
Cinnamic Acid	3,510 mM

Figure S3. IC50 of TAC assay of ripe and unripe *Annurca* apple samples. (Top) Flesh samples, Peel samples and Core samples. (Bottom) Standards for positive control.

	EC50 FRAP Assay	
	Unripe	Ripe
Flesh	1,436 mM	1,007 mM
Peel	1,019 mM	0,5643 mM
Core	1,926 mM	1,223 mM

	EC50 FRAP Assay
Catechin	1,106 mM
Quercetin	1,086 mM
Caffeic Acid	0,965 mM
Epicatechin	0,996 mM
Cholorgenic Acid	1,129 mM
Coumaric Acid	1,333 mM
Cinnamic Acid	2,053 mM

Figure S4. IC50 of FRAP assay of ripe and unripe *Annurca* apple samples. (Top) Flesh samples, Peel samples and Core samples. (Bottom) Standards for positive control.

	EC50 ABTS Assay	
	Unripe	Ripe
Flesh	1,138 mM	0,967 mM
Peel	0,750 mM	0,610 mM
Core	1,246 mM	1,002 mM

	EC50 FRAP Assay
Catechin	1,104 mM
Quercetin	1,334 mM
Caffeic Acid	0,947 mM
Epicatechin	1,302 mM
Cholorgenic Acid	1,395 mM
Coumaric Acid	1,476 mM
Cinnamic Acid	1,648 mM

Figure S5. IC50 of ABTS assay of ripe and unripe *Annurca* apple samples. (Top) Flesh samples, Peel samples and Core samples. (Bottom) Standards for positive control.
